# An unusual case of gender-associated mitochondrial DNA heteroplasmy: the mytilid *Musculista senhousia *(Mollusca Bivalvia)

**DOI:** 10.1186/1471-2148-7-S2-S7

**Published:** 2007-08-16

**Authors:** Marco Passamonti

**Affiliations:** 1 Department of "Biologia Evoluzionistica Sperimentale", University of Bologna, Bologna, I-40126, Italy

## Abstract

**Background:**

Doubly Uniparental Inheritance (DUI) represents the most outstanding exception to matrilinear inheritance of mitochondrial DNA (mtDNA), typical of Metazoa. In a few bivalve mollusks, two sex-linked mtDNAs (the so-called M and F) are inherited in a peculiar way: both daughters and sons receive their F from the mother, whereas sons inherit M from the father (males do not transmit F to their progeny). This realizes a double mechanism of transmission, in which M and F mtDNAs are inherited uniparentally.

DUI systems represent a unique experimental model for testing the evolutionary mechanisms that apply to mitochondrial genomes and their transmission patterns as well as to mtDNA recombination.

**Results:**

A new case of DUI is described in *Musculista senhousia *(Mollusca: Bivalvia: Mytilidae). Its heteroplasmy pattern is in line with standard DUI. Sequence variability analysis evidenced two main results: F haplotypes sequence variability is higher than that of M haplotypes, and F mitochondrial haplotypes experience a higher mutation rate in males' somatic tissues than in females' ones. Phylogenetic analysis revealed also that *M. senhousia *M and F haplotypes cluster separately from that of the other mytilids.

**Conclusion:**

Sequence variability analysis evidenced some unexpected traits. The inverted variability pattern (the F being more variable than M) was new and it challenges most of the rationales proposed to account for sex-linked mtDNA evolution. We tentatively related this to the history of the Northern Adriatic populations analyzed. Moreover, F sequences evidenced a higher mutation level in male's soma, this variability being produced de novo each generation. This suggests that mechanisms evolved to protect mtDNA in females (f.i. antioxidant gene complexes) might be under relaxed selection in males. Phylogenetic analysis of sex-linked haplotypes confirmed that they have switched their roles during the evolutionary history of mytilids, at variance to what has been observed in unionids. Consequently, reciprocal monophyly of M and F lineages got easily lost because of role-reversals and consequent losses of M lineages, as already observed in Mytilus.

## Background

Metazoan mitochondrial DNA is known to be usually transmitted by matrilinear inheritance [1]. Transmission of paternal DNA is episodic in plants and animals, but some bivalve mollusks show two highly divergent gender-associated mitochondrial genomes (the so-called F and M mitochondrial genomes), both transmitted to the progeny [2-10]. Actually, in the *Mytilus edulis *species complex (i.e. *Mytilus edulis*, *M. galloprovincialis*, *M. trossulus*) the co-inheritance of the female and the male mitochondrial genomes have been largely demonstrated and, in more detail, both daughters and sons get the F genome from the mother, whereas sons only inherit and transmit the M genome of the father. This peculiar mtDNA inheritance pattern is known as Doubly Uniparental Inheritance (DUI; [4,5]) or gender-associated inheritance [2,3]. Although DUI was only directly demonstrated in *Mytilus *by crossing experiments (*i.e*. tracing transmission of paternal and maternal mtDNA haplotypes to sons and daughters), the occurrence of distinct gender-associated mtDNA types in a given population is considered as a sound evidence of it, since other possible explanations are not obvious [2].

### Genetics and evolution of DUI systems

Doubly Uniparental Inheritance represents a unique system to test for evolutionary forces acting on mitochondrial genomes: actually, analyses on the *Mytilus *complex showed that mtDNA evolves faster in mussels than in other metazoans [11,12] and this has been related to relaxed selective constraints experienced by DUI systems of inheritance [13]. Moreover, studies on *Mytilus *and *Pyganodon *showed that the M lineage evolves faster than the F one ([6,14], and references therein). Authors suggested several explanations for this: higher rate of M mtDNA replication during early male embryo development and spermatogenesis; free-radical damage in sperm; positive selection; or effects of the smaller population size of the M genome (see [13] for details). The analysis over a large part of the mitochondrial genome of the DUI species *Tapes philippinarum *(Bivalvia Veneridae), while confirming an overall higher mutation rate in M than in F mtDNA, showed that not all M-type genes have levels of mutation significantly higher than F-type, thus suggesting that each gene might experience different selective constraints [15]. Furthermore, tests of neutrality have been carried out comparing the M and F mitochondrial genomes, but obtained data are quite contrasting to date [13-15]. Actually, evidences of both neutral and non-neutral evolution have been found in different *Mytilus *taxa and populations, results that were considered in line with a nearly neutral model of molecular evolution (see discussion).

Another trait of Doubly Uniparental Systems is the mitochondrial heteroplasmy of male somatic tissues (F and M genomes are present together); however, the ratio of M vs F genomes may be very different when considering different species and tissues. For instance, in *M. edulis *males, all somatic tissues are largely dominated by the F genome, but, while adductor muscle and digestion glands always tested positive for the M genome, other analyzed male tissues were either positive or negative through basic PCR assay [16]; in contrast with *Mytilus*, *T. philippinarum *showed a strong predominance of M mtDNAs in somatic tissues [8]. However, there is no detailed study based on sensitive techniques, such as quantitative PCR, that demonstrate differences in amounts in F and M types in somatic tissues and discards effects due to primer affinity to the annealing sites.

### Distribution and phylogenetics of DUI

DUI was found to occur in additional mytilid species (*M. californianus*, *Geunkesia demissa*), as well as in several species of Unionidae (fresh water mussels; see [9], and references therein), and in the venerid *T. philippinarum*, thus evidencing that DUI occurs in phylogenetically distant families and suggested that it might be widely distributed among bivalves [8,15]. The discovery of DUI in three different bivalve clades might be taken as evidence that gender-associated heteroplasmy appeared independently at least three times in Bivalvia (respectively in Mytilidae, Unionidae and Veneridae), and therefore it could be considered as a derived character (apomorphy); however, the alternative hypothesis (i.e. that gender-associated heteroplasmy is ancestral in bivalves) appeared to be more sound [7]. If the latter is true, the splitting of the two sex-linked mitotypes should date back to the origin of Bivalvia; we could then expect that all M and F mitochondrial DNA would form two independent clusters, regardless of the species they come from, and this is not the case. This assumes that M- and F-type mtDNAs, once established, never switched their roles, but it has been observed, however, that in a few *Mytilus *male specimens, sperm carried F-type molecules rather than M-type molecules (homoplasmic males). This was taken to indicate that F lineages might pass to sperm and substitute M lineages from time to time, thus becoming new M-types ("masculinization" of F types). Very recently, this hypothesis became controversial, since, by re-analyzing previously obtained data by [17], no evidence for mussel males lacking M mtDNAs was found. Authors also suggested that a recombination event in the control region between M and F mtDNAs might be the cause of role-reversal and this would have allowed F-like mtDNA to invade paternal germ line [18]. In any case, whatever the mechanism would be, still phylogenetic data of DUI sex-linked haplotypes evidenced that role reversals actually happened in the past, since M and F lineages lost their reciprocal monophyly within Bivalvia; however, a lot is still to know on how this would have happened.

If we accept that "masculinization" did actually act in bivalve mtDNA history, then DUI might still be an ancestral bivalve character (plesiomorphy), although periodical "role-reversal" events effectively reset the time of divergence between gender-associated genotypes ([7] and references therein). Moreover, if DUI is plesiomorphic among Bivalvia, standard uniparental inheritance commonly observed in non-DUI bivalves should be considered as derived from it; uniparentality would therefore likely be to occur through the disruption of DUI molecular mechanisms, as it has been observed in *Mytilus *hybrids [19,20].

In recent years, we started a wide screening of bivalve species belonging to different families. Although several species did not show detectable heteroplasmy, the mytilid species *Musculista senhousia*, recently introduced by accident in the Adriatic Sea [21], showed heteroplasmy congruent with a DUI model of mitochondrial inheritance. This paper deals with its description and the mtDNA sequences were also analyzed in order to obtain hints on molecular evolution of these mitochondrial genomes, as well as to add to the phylogenetic reconstruction of DUI among bivalves.

## Results

### Germline mtDNAs

Light microscope screening of *M*. *senhousia *spawned gametes revealed that both spermatozoa and eggs were fully mature and alive (this has been assessed both by visual inspection and by fecundation attempts that invariably produced viable embryos); also no visible contamination from somatic cells was detected. Therefore, spawning induction via hydrogen peroxide stimulation proved to be a powerful and suitable method to obtain pure gamete fractions of Musculista. However, this method showed to better stimulate males than females, so that nothing was inferred about the real sex ratio of M. senhousia samples; for this reason, no statistical test on the issue is reported in this paper. A more in-depth sex ratio analysis using specific crosses is now in course in our Lab, in line to what has been done in Mytilus [17].

A total of 1394 base pairs (bp) of mtDNA have been unambiguously sequenced from gametes, namely 394 bp of cob gene, 600 bp of cox1 and 400 bp of rrnL gene. 13 cob sequences (7 females and 6 males, [GenBank: AY570019–AY570031]), 20 cox1 sequences (10 females and 10 males, [GenBank: AY570032–AY570051]) and 14 rrnL sequences (9 females and 5 males, [GenBank: AY570052 – AY570065]) were obtained. Results were quite clear-cut, since, invariably, the sequences pertained to two, highly divergent sex-linked groups, in line to what is expected for DUI; no gamete sample appeared to carry haplotypes of the opposite sex.

**Figure 1:**
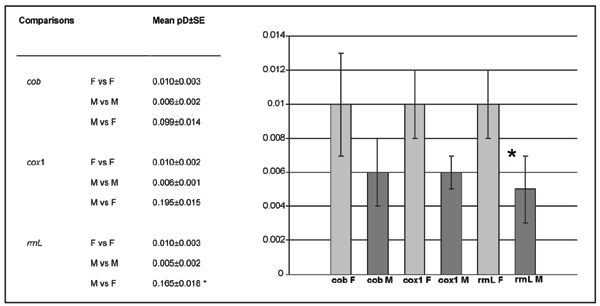
**Sequence variability from gametes**. Mean values and graph of sequence variability (p-distance) of *M. senhousia *sex-linked haplotypes obtained from gametes. Mean and standard error values of nucleotide p-distances are reported for *cob*, *cox1 *and *rrnL *mitochondrial genes. Levels of significance were obtained by random resampling. F, female haplotypes in eggs; M, male haplotypes in sperm; pD, nucleotide p-distance; SE, standard error; *, significant.

The level of divergence between F and M mitotypes was fairly high, the two groups being distinguishable by 28, 90 and 59 fully diagnostic sites, for cob, cox1 and rrnL, respectively; mean p-distance values obtained either within or between sex-linked haplotypes are reported in Fig. 1.

### Somatic MtDNAs

A total of 133 *cob *clones [GenBank: AY614613–AY614702; DQ141817–DQ141859] obtained from 4 females and 8 males were analyzed. While females were invariably homoplasmic for the F mitotype, males showed heteroplasmy fully in line with DUI, but with some peculiarities: the total of 48 sequences obtained from the foot muscle were invariably of the female type, while, out of 43 sequences obtained from the adductor muscle, 12 were of the M type and 31 of the F type. This depicted a situation similar to what has been found in *Mytilus *[16,22,23], but different from that of *T. philippinarum*, in which a comparable experimental approach gave a majority of M clones in the male soma [8]. It was also observed that in males of *M. senhousia *the level of M types in the adductor muscle (*i.e*. percentage of M clones obtained) ranges from 0% to 50%, when considering single specimens, but it is unclear whether this is a real characteristic of *M. senhousia *males, or just a sampling bias. In any case, this observation has to be taken as preliminary only, and more specific experiments on the issue are planned using Real-Time PCR approach.

### Sequence variability

**Figure 2:**
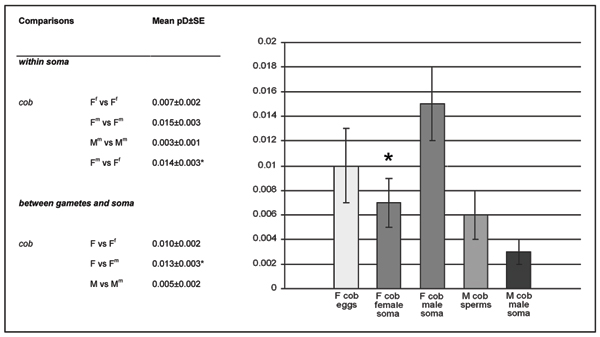
**Sequence variability from somatic tissues**. Mean values and graph of sequence variability (p-distance) of *M. senhousia *sex-linked haplotypes obtained from somatic tissues and comparisons with the gonads. Mean and standard error values of nucleotide p-distances are reported for *cob *mitochondrial gene. Levels of significance were obtained by random resampling. F, female haplotypes in eggs; M, male haplotypes in sperm; pD, nucleotide p-distance; SE, standard error; *, significant.

Analysis of sequence variability has been performed comparing mitochondrial haplotypes within female and male gametes, within soma and between gametes and soma. Values and graphs are reported in Fig. 1 and Fig. 2. When considering mtDNA obtained from gametes, the variability of F is always greater than that of M sequences for all analyzed genes, at variance with previous reports on DUI systems, in which the M genomes usually show higher variability (see discussion below). However, the difference is statistically significant for the *rrnL *gene only (Fig. 1).

Focusing on somatic mitochondrial DNA variability, F haplotypes detected in male soma (referred to as F^m^) appeared to be significantly more variable than the ones detected in the female soma (here referred to as F^f^; Fig. 2).

Comparisons between gonad and somatic haplotypes gave contrasting results: while F^f ^somatic haplotypes were not significantly more variable than F haplotypes from eggs (F), F^m ^somatic haplotypes were significantly more variable than haplotypes from eggs (F). Finally, male haplotypes from sperm (M) appeared to be significantly more variable than the M^m ^from the somatic tissue of males (Fig. 2).

Fisher's exact test (which is a test for positive selection), performed on the *cob *and *cox1 *protein coding genes, was not significant for all analyzed genes (Table 1). This, according to the rationale of the test, revealed that a selection against nonsynonymous mutations is working in *M. senhousia*, i.e. purifying selection is occurring within M and F sequences. However, nonsynonymous mutations are constantly more in M genes, thus suggesting that purifying selection might be stronger in the F mitochondrial genome.

**Table 1:** Fisher's Exact Test of positive selection.

**Gene**		**Synonymous**	**Nonsynonymous**	**Fisher's exact test**^α^
*cob*	F	10	0	0.4923
	M	2	4	0.8432
				
*cox1*	F	17	3	0.5339
	M	10	5	0.7363

Polymorphic sites (synonymous and nonsynonymous mutations) within *cob *and *cox1 *genes of F- and M-type sequences and test of positive selection performed by the one-tailed Fisher's Exact test. Pairwise comparisons within each group sequences (*i.e*. FvsF and MvsM) have been performed. All comparisons gave non-significant values, thus indicating purifying selection. Only obtained average P-values have been reported here for brevity.

^α ^= Average P-value obtained from one-tailed Fisher's exact test.

Moreover, McDonald and Kreitman's test of neutrality indicated no significant deviations from neutrality between M and F sequences (Table 2).

**Table 2:** McDonald and Kreitman test of neutrality.

***Gene***				
		**Substitutions**		
		
		**Fixed**	**Polymorphic**	**Probability**
*cob*	Nonsynonymous	6	4	
	Synonymous	26	12	0.7118
				
*cox1*	Nonsynonymous	22	8	
	Synonymous	81	27	1.000

McDonald and Kreitman test of neutrality between M- and F-type protein sequences. Number of fixed and polymorphic sites (synonymous and nonsynonymous substitutions) between F- and M-type *cox1 *sequences and probabilities from contingency tests are also reported. Significant probability levels as in table 1.

### Phylogenetic analysis of *Musculista*

**Figure 3:**
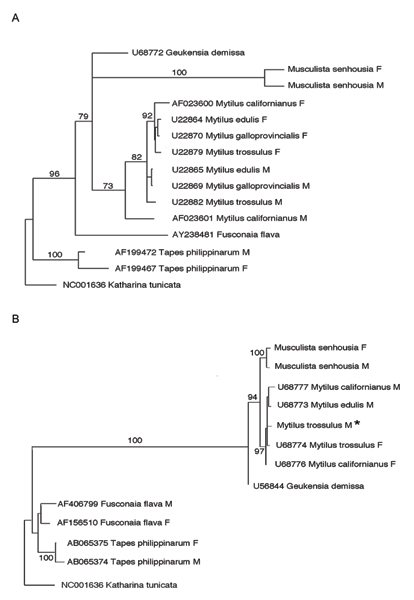
**Phylogenetic inference of DUI-related mitochondrial types**. Maximum Likelihood trees based on *rrnL *(A) and *cox1 *(B) mitochondrial genes, showing phyletic relationships of *Musculista senhousia *sex-linked haplotypes to other DUI species (*Mytilus *spp., *Geukensia demissa*, – Mytilidae; *Fusconaia flava *– Unionidae; *Tapes philippinarum *– Veneridae). Figures above branches indicate bootstrap values (100 replicates). Whenever available, GenBank accession numbers are reported on taxon labels. * *Mytilus trossulus *M *cox1 *sequence is not available in GenBank and it has been obtained from [52].

A phylogenetic analysis has been performed on both *rrnL *and *cox1 *genes, to ascertain origin and phyletic relationships of the *Musculista *sex-linked haplotypes (Fig. 3). *Cob *was not used for this analysis because appropriate Genbank comparison were lacking. Sequences were chosen in order to once represent each DUI family (*Fusconaia flava *for Unionidae and *Tapes philippinarum *for Veneridae), but a wider sampling of Mytilidae has been added to the analysis (*Mytilus edulis*, *M. galloprovincialis*, *M. trossulus*, *M. californianus *and *Geukensia demissa*). Whenever available in GenBank, both M and F-type sequences were included in the analysis. Pertinent information on sequences obtained from GenBank is reported in Fig. 3.

Maximum Likelihood trees are reported in Fig. 3A and 3B for *rrnL *and *cox1*, respectively. The hierarchical likelihood ratio test using Modeltest [24] indicates that the best scored likelihood model for the *cox2 *and *rrnL *datasets is the Transversion Model (TVM, variable base frequencies, variable transversions, transitions equal) plus Gamma (*cox2*, Γ = 0.3740; *rrnL*, Γ = 1.3030). Maximum parsimony produce the following trees: *cox2*, tree length, 1316, Consistency Index, 0.581; *rrnL*, tree length, 930; Consistency Index, 0.713. Both genes produced tree topologies and bootstrap values that were similar to that of Maximum Likelihood, so that the corresponding trees are not reported for brevity.

As expected, *Fusconaia flava *(Unionidae), *Tapes philippinarum *(Veneridae) and Mytilidae form three well-supported clusters; within the Mytilidae, *Musculista *haplotypes always cluster together, but invariably outside the *Mytilus *branch (either M or F). This is true also for the mytilid *Geukensia demissa*. Bootstrap values are very high, strongly supporting the obtained topologies (Fig 3A and 3B). The comprehensive Mytilidae pattern appears somewhat mixed, either with haplotypes clustering in a sex-specific way (*Mytilus*) or haplotypes clustering in a species-specific way (*Musculista*), thus suggesting a different situation from that of Unionidae (see [9]).

## Discussion

### Tissue distribution of sex-related mtDNAs in *Musculista*

This paper provides first evidence for a new Doubly Uniparental Inheritance case of mitochondrial DNA in the mytilid *M. senhousia*. The heteroplasmic pattern obtained is in line with other DUI systems of mtDNA inheritance: sperm and eggs carry two different mitochondrial haplotypes, M and F respectively, so that we can assume that two different mitochondrial genomes are passed to progeny.

Tissue distribution of F and M haplotypes in *Musculista *appears in line with other mytilid species, although M haplotypes were relatively rare in the male soma. The distribution of the M mitotype in adult DUI males allows making important predictions on the mechanisms transmitting M mitochondria to progeny. It is trivial that the egg carries many more mitochondria than the spermatozoon; it has also been observed that sperm mitochondria enter the egg during fertilization ([25]; personal observations on *Tapes philippinarum*); for this, it is reasonable to postulate that, in the zygote, F mitochondria, derived from the egg, must overwhelm the M ones, inherited from the spermatozoon. Nonetheless, M mitochondria have to find their way to blastomer 4d, which is known to give rise to germ line [26]; this may happen in two ways: the first is that M type should undergo several replications during the early male embryo cleavage, so that M mitochondria will have a good chance to be included in the 4d blastomer [23]; the second, is that sperm mitochondria are actively segregated to the 4d blastomer itself. In both cases, the M mitochondria displace F ones during testis development and, if the first scenario is true, then we may expect a large quantity of M mitochondria in the male somatic tissues of adults, while, if the second is true, we may expect no or few M genomes in the soma.

The second scenario seems to fit better the case of *Musculista*: M haplotypes are extremely rare in male somatic tissues of this species, so that we can assume that M mitochondria are actively segregated to germ line. Moreover, recent observations of the cytological behavior of sperm mitochondria in early embryo stages showed that the second scenario seems also true for *Mytilus edulis*: using *in vivo *mitochondrial staining, it was shown that sperm-derived mitochondria are actively segregated to blastomer 4d in the early cleavages of male embryos [27]. It must be noted, however, that M mitochondrial DNAs are also detectable in specific *Mytilus *somatic tissues (this is also confirmed in *Musculista*), so that the M segregation to blastomer 4d must not be a highly selective mechanism. Actually, studies on M-type expression through RT-PCR in *Mytilus edulis *showed that M mitochondrial DNA produce functional mRNAs in about 50% of the analyzed male somatic tissues [28].

### Molecular evolution of sex-related mtDNAs in *Musculista*

Although the discovery of sex-related heteroplasmy is not surprising for a mytilid, one trait of *Musculista *DUI system seems unique: F haplotypes appear more variable than M for the analyzed genes, and this contrasts with all previously described DUI systems ([6,14], and references therein). This unexpected character holds true for all three analyzed genes, either coding for proteins or for rRNA, although only one comparison appeared to be statistically significant (*rrnL *gene, see Fig. 1). These results suggest that higher variability could be a general feature of the whole *M. senhousia *mitochondrial genome, but the ultimate demonstration will be available only when the complete M and genomes are sequenced (its completion is now in progress in our lab). At any rate, this finding challenges a major DUI tenet: in hitherto known DUI systems, three main mechanisms have been proposed to account for a higher sequence variability of M vs. F mtDNA, namely enhanced replication rate during spermatogenesis, free-radical damage to sperm, or the effect of the smaller population size of the M genome (see [13] for details). The DUI of *M. senhousia *is likely to experience the same conditions as those of the other DUI systems; therefore, this mechanism cannot account for the reverse variability pattern of *Musculista*, and other explanations have to be sought for.

Let us first consider the model proposed by "Stewart *et al*." [13], and envisage what it would predict when applied to *M. senhousia*. The "Stewart *et al*." hypothesis differentiates three selective arenas for mitochondria, namely the somatic cell line, the female germline, the male germline and it assumes that there might be some tradeoffs in terms of optimal functioning. According to this hypothesis, in DUI species the M genome has to function in the male germline only, while F genome has to function in soma (of both sexes) as well as in the female germline. The outcome is that the F genome is more constrained and its nucleotide variability should be lower than that of the M genome. Applying this model to *M. senhousia*, the outcome should not be different, so other hypotheses have to be considered.

An alternative hypothesis is that the M mitochondrial genome in *M. senhousia *might undergo a stronger selection pressure than that on the F genome, and this would lower the level of variability of the M genome. Although this seems to be in line with the results of the Fisher's exact test, which supported purifying selection in *M. senhousia *sequences, the fact that M mitochondrial genes (although less variable) showed proportionally more nonsynonymous mutations than F would contradict this (Table 1). Moreover, the McDonald and Kreitman test shows that M and F types have diverged during a long period of time in a neutral way, and this would also contradict the above-mentioned rationale. In any case, we should keep in mind that the results of this test must be taken with extreme caution, being strongly affected by population bottlenecks, which are known to affect the reliability of it [29]. Incidentally, this is what we know has happened with *M. senhousia *in the Adriatic Sea, since commercial ships likely introduced few specimens in it [21]. Moreover, more information on effective population size for M and F genomes is considered very important to tell apart a nearly neutral situation from a traditional theory of positive selection [30,31]. This might account for contrasting results when applying the test to different populations/species.

Following the rationale of [16] and the abovementioned observations, we cannot exclude that *M. senhousia *mtDNA evolved under a nearly neutral model of evolution, in which slightly deleterious mutations can still be present, especially in males, where some degree of relaxed selection might permit their short-term existence. But if this is true, still we have to explain why males showed an overall lower level of mutation. Evidences have been recently proposed that mtDNA would be largely affected by repetitive events of selective sweep; according to this, mtDNA appears to evolve in agreement with the recurrent fixation of advantageous mutations leading to frequent loss of variability at linked loci, a process named "genetic draft" ([32], and references therein). If this is applied to the *M. senhousia *mitochondrial genome, then we can hypothesize that a recent selective sweep event might have reduced dramatically the variation of M mtDNA, and this might be related to the new environmental conditions of the recently introduced Northern Adriatic populations. This hypothesis needs to be tested comparing complete mtDNA sequences of Adriatic specimens to samples from the original range of the species, which is not possible at the moment.

Another hypothesis that could be considered is that the low numerical level of M mitotypes in the male soma of *M. senhousia *might reduce the possibility of metabolic damages of M-type mtDNA during early replications. However, this mechanism would work only if, each generation, a representative sub-sampling of the total mtDNA variability is transmitted to progeny. Although metabolic damage of somatic mitochondrial DNA has been demonstrated, germline mitochondria appear to be somehow protected, in order to keep the integrity of the mitochondrial genome [33]. If this is true, mitochondria passing to progeny belong to an organelle population that is largely distinct from somatic one, and mitochondria accidentally remaining in somatic tissues are most likely "dead ends" [34,35] (an analogy to germline cells in metazoans is evident). This also suggests that there might be some mechanism that allow recognition and segregation of germline mitochondria, and this might interface with the described cytological behavior of sperm mitochondria in the early embryo stages of *M. edulis *[27], which evidenced that M mitochondria are actively segregated to germline cells.

A last hypothesis could take into account the heavy bottleneck that *M. senhousia *could have passed when accidentally carried to the Northern Adriatic Sea [21]. If we hypothesize that in the founding population males were much fewer than females, this would likely result in lower variability in the M genome. This scenario seems appropriate to explain the particularities of the *Musculista *DUI system: this species has been possibly introduced by accident to the Northern Adriatic Sea with the clam *Tapes philippinarum*, imported for acquaculture in 1986, and is known to experience dramatic population fluctuations (see  for details) that might affect the levels of variability of both mitochondrial genomes. Unfortunately, this has to be taken as a non-testable hypothesis, since no data are available on the effective number of individuals introduced in the Adriatic Sea. Also, we have to mention that to better test for this scenario, a wide population sample from the original range of *M. senhousia *– in which narrow bottlenecks are unlikely to have occurred in the past – would be needed. Unfortunately, at the moment, we are not able to perform such a test.

The nature of the DUI system, which has lead to the presence of distinct M and F mitochondria in somatic tissues, gives us the opportunity to compare levels of variability of mtDNAs both within soma and between soma and gonads. It has to be mentioned that, when comparing gonad vs somatic mtDNAs, the different sequencing protocols (from PCR products or cloning, see Methods section for details) might affect our comparisons: actually, given that gonad mtDNAs have been sequenced directly without cloning, low frequency variants would not be detected among them. As a consequence, we could expect that our variability estimates from gonads are lower than the real. However, the results showed here indicate that estimated variability of gonads is always higher than in soma (see Fig. 2), so that the real gonad variability can only be higher. For this reason, we think that the method of analysis did not significantly affect our tests.

Variability analyses of somatic F haplotypes gave additional unexpected and interesting results: F mtDNAs isolated from the male soma (F^m^) are significantly more variable than those from the female soma (F^f^). Moreover, somatic F haplotypes of males (F^m^) appeared to be 2-fold more variable than female gonad haplotypes (F). The overall pattern of variability suggests that there might be a higher level of mutation when F mtDNAs are in a male than in female, and that increased variability has to be produced *de novo *in males each generation.

In the literature there is substantial evidence that male-inherited DNA (*f.i*. Y-linked neutral sites) evolves at a higher rate than female-inherited DNA (*f.i*. X-linked neutral sites), and this is also true for most of the M and F mtDNAs known in DUI systems (except for *M. senhousia*, incidentally; see above). This has been related to a greater number of cell divisions during the production of spermatozoa and the consequent production of new mutations due to replication errors (see [36] and references therein). However, this is not true for *M. senhousia *F mitochondria, which showed higher variability in male soma, because they both derive from the egg in males and females. Increasing evidence indicates that replication-independent factors may be responsible for this; actually, a higher antioxidant gene-expression and a lower oxidative damage of mitochondria of females has been observed and related to estrogen production in rats [37]. Moreover, this trait fits well within evolutionary predictions: antioxidant gene complexes, evolved to protect mitochondria from oxidative damages, might be under relaxed selection in non-DUI males, being male mitochondria dead-ends. It seems sound to speculate that an analogous mechanism might also work in *M. senhousia*: although we cannot exclude that selection over antioxidant gene complexes might be stronger in a DUI male, which is not a mitochondrial dead end, we have to remember that M mitochondria have to be fully functional in sperms only (see [13]), and this might still have allowed some degree of relaxed selection over antioxidant complexes in DUI males.

### Phylogenetic analysis of sex-linked *M. senhousia *mtDNAs

Phylogenetic analysis of *M. senhousia *sex-linked haplotypes revealed that both M and F cluster separately from that of the other mytilids. The situation here depicted shows that mytilid M and F mtDNAs do not pertain to two different lineages, regardless the species they come from, as it happens in *Mytilus *and in unionids [9,38]. Since it is generally accepted that DUI predates the origin of mytilids, we are allowed to confirm that "masculinization" (*i.e*. role-reversal) was present during the whole evolutionary history of mytilids, not only in *Mytilus sensu stricto*: our data on *M. senhousia *strongly suggest that, during the lineage leading to it, old M-types got lost once or more times. As a consequence, it is trivial that, if role-reversals and consequent losses of M lineages do occur, then we have to expect that the reciprocal monophyly of M and F lineages got easily lost during the evolutionary history of mytilids. As already mentioned, role-reversals of the M and F genomes have been observed in *Mytilus*, both as direct evidence in laboratory crosses [5,39], and in natural populations [40,41], with "masculinization" being more common than "feminization", although those observations has been recently doubted [18].

## Methods

### Sample collection

*M. senhousia *specimens from Venice Lagoon (Italy) were used for analysis. About 50 specimens were stimulated to emit sperm or eggs in seawater added with hydrogen peroxide, according to [42]. As soon as the treated seawater was removed and the mussels introduced in single recipients with clear seawater, some of them started emitting sperm or eggs. Each emission was analyzed by light microscopy to sex the specimens, as well as to detect eventual contamination by somatic cells, and a total of 10 sperm and 10 egg samples were further analyzed. Gametes were then collected after a gentle centrifugation (3000 × *g*) and seawater removed. Separately, two samples of somatic tissue (terminal tip of the foot and adductor muscle) were dissected under light microscope to avoid contamination with gonads as well. Gametes and somatic tissues were immediately frozen and stored at -20°C for subsequent analyses.

### DNA isolation and PCR

Total genomic DNA was extracted from each gamete sample using the DNeasy Tissue Kit (Qiagen).

Partial sequences of Cytochrome b (*cob*), Cytochrome oxidase subunit I (*cox1*), and mitochondrial ribosomal large subunit RNA (*rrnL*) were amplified and directly sequenced without cloning, as described in [15]. The primers were: cobR (5'-GCR TAW GCR AAW ARR AAR TAY CAY TCW GG-3') and cobF (5'-GGW TAY GTW YTW CCW TGR GGW CAR AT-3') for *cob *(designed by J. L. Boore), HCO2198 and LCO1490 for *cox1 *[43] and 16Sbr and 16SarL for *rrnL *[44]. Sequencing reactions were performed on both strands with BigDye Terminator Cycle Sequencing Kit according to supplier's instructions (Applied Biosystem) in a 310 Genetic Analyzer (ABI) automatic sequencer.

Total genomic DNA was also obtained from tip of foot (4 males, 4 females) and adductor muscle (4 males). The partial sequence of *cob *was amplified from the abovementioned samples. We used *cob *for three main reasons: (1) the relatively short tract to be sequenced; (2) the high quantity of informative sites; and (3) the fact that *cob *primers, being quite degenerate, made us more confident that we would not lose part of the overall variability of the gene because of incidental primer annealing failures in PCR. This is a critical point, since lacking of primer annealing might affect our estimates, as well as bias them towards one of the two sexes. Choosing sex-specific primers would not therefore solve the issue, since eventual primer failures would not be excluded in any case. We therefore think that the use of two non-degenerated primers would be less good than using *n*-fold degenerate ones (i.e. 2 × *n *different primers in the same PCR reaction).

Amplified fragments were subsequently cloned using the pGEM-T Easy Vector System Kit (Promega). Clones were then sequenced on both strands using M13 universal primers.

### Sequence analysis

Sequence data from this article have been deposited with the EMBL/Genbank Data Libraries [GenBank: AY614613–AY614702; DQ141817–DQ141859].

Sequences were aligned using the Clustal algorithm of the MT Navigator PPC software (Applied Biosystems). Alignments were then edited manually, taking into account aminoacid sequences (*cob*, *cox1*), as well as potential secondary structures (*rrnL*).

To analyze sequence variability, pairwise p-distances, their mean values and standard errors (by the bootstrap procedure) were obtained within each group (from eggs, sperm and soma, respectively). The use of a p-distance estimator has been preferred for simplicity, because we did not want to introduce any model of DNA substitution, which might have an influence in the performed tests, and because the use of p-distance estimators has been already used also in earlier literature [15]. Moreover, we calculated the mean number of sequence differences within each group (*d*_*n*_) and their pairwise differences (Δ˙
 MathType@MTEF@5@5@+=feaafiart1ev1aaatCvAUfKttLearuWrP9MDH5MBPbIqV92AaeXatLxBI9gBaebbnrfifHhDYfgasaacH8akY=wiFfYdH8Gipec8Eeeu0xXdbba9frFj0=OqFfea0dXdd9vqai=hGuQ8kuc9pgc9s8qqaq=dirpe0xb9q8qiLsFr0=vr0=vr0dc8meaabaqaciaacaGaaeqabaqabeGadaaakeaacuqHuoargaqgaaaa@2E32@ = *d*_α _- *d*_β _; data not shown). We then performed a random resampling of each group to obtain the null distribution of Δ and, if this did not include 0 (with α = 0.05), then the observed values were considered significantly different. G. Bertorelle (University of Ferrara, Italy) suggested the test and developed the software, which is available from the Author.

Moreover, in order to analyze sequences for neutrality, we performed tests based on inferred protein polymorphism of the *cob *and *cox1 *genes. The number of polymorphic sites within M and F *cob *and *cox1 *(either showing synonymous or non-synonymous mutations) was obtained, and a test of positive selection was performed by a one-tailed Fisher's exact test [45], as implemented by MEGA2 [46]. This test is considered more appropriate than the Z-test when the number of nucleotide substitutions per sequence is small [47], which is the case for our comparisons.

Comparisons between *cox1 *M- and F-types were performed by the test of McDonald and Kreitman [48], as implemented in DnaSP 4.0 [49]. The test is based on the observation that, under neutrality, the ratio of non-synonymous to synonymous fixed substitutions between M and F types should be the same as the ratio of non-synonymous to synonymous polymorphism within types.

Phylogenetic analysis of DUI distribution was performed using partial *rrnL *and *cox1 *sequences, whenever available in GenBank. Beside *M. senhousia*, the following additional species were included in the analysis: *Mytilus edulis*, *M. galloprovincialis*, *M. trossulus*, *M. californianus*, *Geukensia demissa *(Mytilidae), *Tapes philippinarum *(Veneridae) and *Fusconaia flava *(Unionidae), as a representative of the many DUI species of this family (see [9]). Sequences of *Katharina tunicata *(Mollusca: Polyplacophora; [50]) were used as outgroups.

Phylogenetic analysis was performed using Maximum Parsimony and Maximum Likelihood approaches, using PAUP* (version 4.0, [51]); likelihood consensus trees have been obtained after 100 bootstrap replicates. Maximum parsimony was obtained with heuristic searching, and the bootstrap consensus tree was obtained after 2000 replicates. Likelihood scores of each DNA substitution model were calculated using Modeltest software [24] and the best-scored model was used for Maximum Likelihood tree reconstructions. Support to the dendrogram was obtained using bootstrap (100 replicates).

## Competing interests

The authors declare that they have no competing interests.

## Authors' contributions

MP performed data collection, sequence alignments and analysis, statistical tests and tree constructions, and wrote the manuscript.

## References

[B1] Birky CW (1995). Uniparental inheritance of mitochondrial and chloroplast genes: mechanisms and evolution. Proc Natl Acad Sci USA.

[B2] Skibinski DOF, Gallagher C, Beynon CM (1994). Mitochondrial DNA inheritance. Nature.

[B3] Skibinski DOF, Gallagher C, Beynon CM (1994). Sex-limited mitochondrial DNA transmission in the marine mussel *Mytilus edulis*. Genetics.

[B4] Zouros E, Ball AO, Saavedra C, Freeman KR (1994). Mitochondrial DNA inheritance. Nature.

[B5] Zouros E, Oberhauser Ball A, Saavedra C, Freeman KR (1994). An unusual type of mitochondrial DNA inheritance in the blue mussel *Mytilus*. Proc Natl Acad Sci USA.

[B6] Liu HP, Mitton JB, Wu SK (1996). Paternal mitochondrial DNA differentiation far exceeds maternal mitochondrial DNA and allozyme differentiation in the freshwater mussel, *Anodonta grandis grandis*. Evolution.

[B7] Hoeh WR, Stewart DT, Saavedra C, Sutherland BW, Zouros E (1997). Phylogenetic evidence for role-reversals of gender-associated mitochondrial DNA in *Mytilus *(Bivalvia: Mytilidae). Mol Biol Evol.

[B8] Passamonti M, Scali V (2001). Gender-associated mitochondrial DNA heteroplasmy in the venerid clam *Tapes philippinarum *(Mollusca: Bivalvia). Curr Genet.

[B9] Hoeh WR, Stewart DT, Guttman SI (2002). High fidelity of mitochondrial genome transmission under the doubly uniparental mode of inheritance in freshwater mussels (Bivalvia: Unionoidea). Evolution Int J Org Evolution.

[B10] Serb JM, Lydeard C (2003). Complete mtDNA s equence of the North American freshwater mussel, *Lapsilis ornata *(Unionidae). Mol Biol Evol.

[B11] Hoeh WR, Stewart DT, Sutherland BW, Zouros E (1996). Cytochrome c oxidase sequence comparisons suggest an unusually high rate of mitochondrial DNA evolution in Mytilus (Mollusca: Bivalvia). Mol Biol Evol.

[B12] Hoffmann RJ, Boore JL, Brown WM (1992). A novel mitochondrial genome organization for the blue mussel, *Mytilus edulis*. Genetics.

[B13] Stewart DT, Kenchington ER, Singh RK, Zouros E (1996). Degree of selective constraint as an explanation of the different rates of evolution of gender-specific mitochondrial DNA lineages in the mussel *Mytilus*. Genetics.

[B14] Quesada H, Warren M, Skibinski DOF (1998). Nonneutral evolution and differential mutation rate of gender-associated mitochondrial DNA lineages in the marine mussel *Mytilus*. Genetics.

[B15] Passamonti M, Boore JL, Scali V (2003). Molecular evolution and recombination in gender-associated mitochondrial DNAs of the Manila clam *Tapes philippinarum*. Genetics.

[B16] Garrido-Ramos MA, Stewart DT, Sutherland BW, Zouros E (1998). The distribution of male-transmitted and female-transmitted mitochondrial DNA types in somatic tissues of blue mussels: implications for the operation of doubly uniparental inheritance of mitochondrial DNA. Genome.

[B17] Saavedra C, Reyero MI, Zouros E (1997). Male-dependent doubly uniparental inheritance of mitochondrial DNA and female dependent sex-ratio in the mussel *Mytilus galloprovincialis*. Genetics.

[B18] Theologidis I, Saavedra C, Zouros E (2007). No evidence for absence of paternal mtDNA in male progeny from pair-matings of the mussel *Mytilus galloprovincialis*. Genetics.

[B19] Rawson PD, Secor CL, Hilbish TJ (1996). The effects of natural hybridization on the regulation of doubly uniparental mtDNA inheritance in blue mussels (*Mytilus *ssp.). Genetics.

[B20] Wood AR, Turner G, Skibinski DOF, Beaumont AR (2003). Disruption of doubly uniparental inheritance of mitochondrial DNA in hybrid mussels (*Mytilus edulis *× **M. galloprovincialis**). Heredity.

[B21] Lazzari G, Rinaldi E (1994). Alcune considerazioni sulla presenza di specie extra Mediterranee nelle lagune salmastre di Ravenna. Boll Malac.

[B22] Stewart DT, Saavedra C, Stanwood RR, Oberhauser Ball A, Zouros E (1995). Male and female mitochondrial DNA lineages in the blue mussel (*Mytilus edulis*) species group. Mol Biol Evol.

[B23] Sutherland B, Stewart B, Kenchington ER, Zouros E (1998). The fate of paternal Mitochondrial DNA in developing female mussels, *Mytilus edulis *: implications for the mechanism of Doubly Uniparental Inheritance of Mitochondrial DNA. Genetics.

[B24] Posada D, Crandall KA (1998). Modeltest: testing the model of DNA substitution. Bioinformatics.

[B25] Longo FJ, Dornfield EJ (1967). The fine structure of spermatid differentiation in the mussel, *Mytilus edulis*. J Ultrastruct Res.

[B26] Verdonk NH, Van Den Biggelaar JAM, Verdonk NH, Van Den Biggelaar JAM, Tompa AS (1983). Early development and the formation of the germ layers. The Mollusca: development.

[B27] Cao L, Kenchington ER, Zouros E (2004). Differential segregation patterns of sperm mitochondria in embryos of the blue mussel (*Mytilus edulis*). Genetics.

[B28] Dalziel AC, Stewart DT (2002). Tissue-specific expression of male-transmitted mitochondrial DNA and its implications for rates of molecular evolution in *Mytilus *mussels (Bivalvia: Mytilidae). Genome.

[B29] Eyre-Walker A (2002). Changing effective population size and the McDonald-Kreitman Test. Genetics.

[B30] Skibinski DOF, Gallagher C, Quesada U (1999). On the roles of selection, mutation and drift in the evolution of mitochondrial DNA diversity in British *Mytilus edulis *(Mytilidae; Mollusca) populations. Biol J Linn Soc.

[B31] Ohta T (1996). The current significance and standing of neutral and nearly neutral theories. Bioessays.

[B32] Bazin E, Glémin S, Galtier N (2006). Population size does not influence mitochondrial genetic diversity in animals. Science.

[B33] Kajander OA, Rovio AT, Majamaa K, Poulton J, Spelbrink JN, Holt IJ, Karhunen PJ, Jacobs HT (2000). Human mtDNA sublimons resemble rearranged mitochondrial genomes found in pathological states. Hum Mol Genet.

[B34] Jansen RP, Barrit JA, Brenner CA, Willadsen S, Cohen J, Shoubridge EA (2000). Germline passage of mitochondria: Quantitative considerations and possible embryological sequelae. Hum Reprod.

[B35] Shoubridge EA (2000). Mitochondrial DNA segregation in the developing embryo. Hum Reprod.

[B36] Whittle CA, Johnston MO (2002). Male-driven evolution of mitochondrial and chloroplastidial DNA sequences in plants. Mol Biol Evol.

[B37] Borras C, Sastre J, Garcia-Sala D, Lloret A, Pallardò FV, Viña J (2003). Mitochondria from females exhibit higher antioxidant gene expression and lower oxidative damage than males. Free Radic Biol Med.

[B38] Curole JP, Kocher TD (2002). Ancient sex-specific extension of the cytochrome c oxidase II gene in bivalves and the fidelity of doubly-uniparental inheritance. Mol Biol Evol.

[B39] Fisher C, Skibinski DOF (1990). Sex-biased mitochondrial DNA heteroplasmy in the marine mussel *Mytilus*. Proc R Soc Lond B Biol Sci.

[B40] Quesada H, Wenne R, Skibinski DOF (1999). Interspecies transfer of female mitochondrial DNA is coupled with role-reversal and departure from neutrality in the mussel *Mytilus trossulus*. Mol Biol Evol.

[B41] Laudokakis ED, Saavedra C, Margoulas A, Zouros E (2002). Mitochondrial DNA variation in a species with two mitochondrial genomes: the case of *Mytilus galloprovincialis *from the Atlantic, the Mediterranean and the Black Sea. Mol Ecol.

[B42] Morse DE, Duncan H, Hooker N, Morse A (1977). Hydrogen peroxide induces spawning in molluscs, with activation of prostaglandin endoperoxide synthtase. Science.

[B43] Folmer O, Black M, Hoeh W, Lutz R, Vrijenhoek R (1994). DNA primers for amplification of mitochondrial cytochrome c oxidase subunit I from diverse metazoan invertebrates. Mol Mar Biol Biotechnol.

[B44] Palumbi S, Martin A, Romano S, McMillan WO, Stice L, Grabowski G (1991). The simple fools guide to PCR.

[B45] Zhang J, Kumar S, Nei M (1997). Small-sample tests of episodic adaptive evolution: A case study of primate lysozymes. Mol Biol Evol.

[B46] Kumar S, Tamura K, Jakobsen IB, Nei M (2001). MEGA2: Molecular Evolutionary Genetics Analysis software.

[B47] Nei M, Kumar S (2001). Molecular Evolution and Phylogenetics.

[B48] McDonald JH, Kreitman M (1991). Adaptative protein evolution at the *Adh *locus in *Drosophila*. Nature.

[B49] Rozas J, Sanchez-DelBarrio JC, Messeguer X, Rozas R (2003). DnaSP, DNA polymorphism analyses by the coalescent and other methods. Bioinformatics.

[B50] Boore JL, Brown WM (1994). Complete DNA sequence of the mitochondrial genome of the black chiton, *Katharina tunicata*. Genetics.

[B51] Swofford DL (2003). PAUP*. Phylogenetic Analysis Using Parsimony (*and Other Methods) Version 4.

[B52] Hoeh WR, Stewart DT, Sutherland W, Zouros E (1996). Multiple origins of gender-associated mitochondrial DNA lineages in bivalves (Mollusca: Bivalvia). Evolution.

